# FHIR-Former: enhancing clinical predictions through Fast Healthcare Interoperability Resources and large language models

**DOI:** 10.1093/jamia/ocaf165

**Published:** 2025-10-13

**Authors:** Merlin Engelke, Giulia Baldini, Jens Kleesiek, Felix Nensa, Amin Dada

**Affiliations:** Institute for Artificial Intelligence in Medicine, University Medicine Essen, Essen, 45131, Germany; Institute of Diagnostic and Interventional Radiology and Neuroradiology, University Medicine Essen, Essen, 45147, Germany; Institute for Artificial Intelligence in Medicine, University Medicine Essen, Essen, 45131, Germany; Institute of Diagnostic and Interventional Radiology and Neuroradiology, University Medicine Essen, Essen, 45147, Germany; Institute for Artificial Intelligence in Medicine, University Medicine Essen, Essen, 45131, Germany; Faculty of Computer Science and Medical Faculty, University of Duisburg-Essen, Essen, 45127, Germany; Department of Physics, TU Dortmund University, Dortmund, 44227, Germany; Institute for Artificial Intelligence in Medicine, University Medicine Essen, Essen, 45131, Germany; Institute of Diagnostic and Interventional Radiology and Neuroradiology, University Medicine Essen, Essen, 45147, Germany; Institute for Artificial Intelligence in Medicine, University Medicine Essen, Essen, 45131, Germany

**Keywords:** Large Language Models (LLMs), Clinical Prediction Tasks, Electronic Health Records (EHR), Fast Healthcare Interoperability Resources (FHIR), Multimodal Data, Hospital Readmission, In-hospital Mortality, Imaging Study Prediction, ICD Code Classification

## Abstract

**Objective:**

To address the challenges of data heterogeneity and manual feature engineering in clinical predictive modeling, we introduce FHIR-Former, an open-source framework integrating Fast Healthcare Interoperability Resources (FHIR) with large language models (LLMs) to automate and standardize clinical prediction tasks.

**Materials and Methods:**

FHIR-Former dynamically processes structured (eg, lab results, medications) and unstructured (eg, clinical notes) data from FHIR resources. The pipeline supports multiple classification tasks, including 30-day readmission, imaging study prediction, and ICD code classification. Leveraging open-source LLMs (GeBERTa), we trained models on 1.1 million data points across ten FHIR resources using retrospective inpatient data (2018-2024). Hyperparameters were optimized via Bayesian methods, and outputs were mapped to FHIR RiskAssessment resources for interoperability.

**Results:**

FHIR-Former achieved an F1-score of 70.7% and accuracy of 72.9% for 30-day readmission, 51.8% F1-score (88.1% accuracy) for mortality prediction, and 61% macro F1-score for imaging study classification. The ICD code prediction model attained 94% accuracy. Performance demonstrated promising performance for readmission and showed scalability across tasks without manual feature engineering.

**Discussion:**

FHIR-Former eliminates institution-specific preprocessing by adapting to diverse FHIR implementations, enabling seamless integration of multimodal data. Its configurable architecture outperformed prior frameworks reliant on static inputs or limited to unstructured text. Real-time risk scores embedded in FHIR servers enhance clinical workflows without disrupting existing practices.

**Conclusion:**

By harmonizing FHIR standardization with LLM flexibility, FHIR-Former advances scalable, interoperable predictive modeling in healthcare. The open-source framework facilitates automation, improves resource allocation, and supports personalized decision-making, bridging gaps between AI innovation and clinical practice.

## Background and significance

The digitalization of healthcare systems has led to an unprecedented availability of electronic health data, providing new opportunities to improve clinical decision-making and patient outcomes. Among the most promising applications of this data is the use of predictive models to forecast clinical outcomes such as disease progression,^1^^,^^2^ hospital readmissions,^3^^,^^4^ and patient survival.^5^^,^^6^ These predictive models can help healthcare providers allocate resources more efficiently, personalize treatment plans, and ultimately improve patient care. However, despite their potential, existing models face several challenges in effectively utilizing the diverse and complex data found in electronic health records (EHR), as well as in transferring and generalizing their performance across different sites.

One of the key challenges in current predictive modeling approaches is the heterogeneity of clinical data.^7^^,^^8^ EHRs contain both structured information, such as lab results and medication histories, and unstructured data, like clinical notes and discharge summaries. Integrating these disparate data types into a cohesive and accurate prediction model remains a significant obstacle for researchers.^9^ Furthermore, many existing models rely on manual feature selection or predefined input types,^10–12^ which limits their scalability and adaptability to new datasets or clinical tasks. This lack of flexibility has slowed the adoption of predictive models across diverse healthcare settings.

Fast Healthcare Interoperability Resources (FHIR) has emerged as a potential solution, providing a standardized format for clinical data exchange and integration. By offering a uniform structure for representing healthcare data, FHIR holds the promise of simplifying data preprocessing and enhancing interoperability.^13^^,^^14^ In recent years, national policies have accelerated the adoption of HL7 FHIR for interoperable EHRs. In the United States, the 21st Century Cures Act Final Rule requires all certified EHR systems to support standardized FHIR-based application programming interfaces (APIs), which enable structured access to patient and population health data. Since 2021, this regulation has made FHIR interfaces widely available among U.S. healthcare providers, thereby improving patient access and data exchange capabilities. In Germany, the ISiK standard (Informationstechnische Systeme in Krankenhäusern), based on the legal mandate of §373 SGB V, defines the required FHIR-based interfaces for hospital information systems. However, despite these standardization efforts, specific implementations of FHIR can differ significantly between institutions,^15^^,^^16^ leading to additional preprocessing burdens and limiting the integration of predictive models across diverse healthcare settings.

To address these limitations, we introduce FHIR-Former, a fully configurable, end-to-end pipeline designed to integrate FHIR resources with encoder-based language models. FHIR-Former is built to handle multiple types of clinical data, including both structured data like lab values and medications, as well as unstructured data such as clinical reports. This dynamic configurability allows FHIR-Former to be applied flexibly to a range of clinical prediction tasks, including survival prediction and 30-day hospital readmission, without requiring manual feature selection. By offering a scalable and adaptable solution, FHIR-Former provides a framework that can be easily applied across different clinical use cases, advancing the automation and flexibility of clinical prediction models.

Machine learning and deep learning models have been widely applied to EHRs to predict various clinical outcomes, and frameworks leveraging FHIR have emerged to standardize and automate these efforts. Several notable works have laid the groundwork for developing more scalable and flexible pipelines. Machine learning and deep learning have been widely used with EHRs to predict clinical outcomes, with FHIR-based frameworks helping to standardize and automate these efforts. Rajkomar et al^10^ developed a deep learning model using structured EHR data to predict survival, readmission, and length of stay, but it required manual feature selection and had limited scalability. Similar challenges were seen in other models relying on manual extraction and large-scale data handling.^11^^,^^12^ Later work incorporated unstructured notes. ClinicalBERT,^17^ for example, pretrained a BERT model on clinical notes and fine-tuned it for readmission prediction, showing transformers’ strength with complex clinical text. Automation has also advanced with AutoML frameworks^18^^,^^19^ that streamline model training. Cardea,^18^ integrates diverse FHIR data types like medications and labs, reducing manual effort and supporting various predictions.

Our proposed pipeline, FHIR-Former, combines these advancements by offering a fully configurable, end-to-end solution that integrates FHIR resources with encoder-based language models for tasks like survival prediction and 30-day readmission. Unlike previous approaches that either required manual feature selection or limited input types, FHIR-Former allows dynamic configuration of input sources, including clinical reports, lab values, and medications from patient history, providing a flexible and scalable alternative for training prediction models. This adaptability makes FHIR-Former well-suited for a range of clinical use cases, expanding on the automation and flexibility offered by prior systems.

## Methods

### FHIR-Former framework

**Figure 1.**
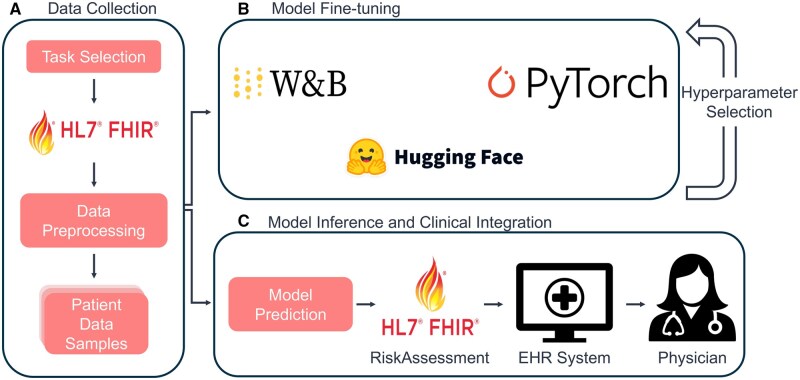
Overview of the FHIR-Former workflow for developing and deploying predictive models. Process A involves task selection and preprocessing of HL7 FHIR-based patient data. Process B fine-tunes models using frameworks like Hugging Face, PyTorch, and Weights & Biases, with hyperparameter optimization. Process C performs model inference and clinical integration: predictions are converted to risk scores and stored as FHIR R4 RiskAssessment resources on the hospital FHIR server, from which the EHR system retrieves and displays them to clinicians.

The FHIR-Former framework has been developed for the purpose of streamlining the training and deployment of models for downstream tasks, leveraging structured EHR formatted according to the HL7 FHIR standard. The FHIR-Former framework operates through a three-stage pipeline (Figure 1). Initially, the system performs task selection and preprocessing of HL7 FHIR-based patient data, extracting and standardizing both structured and unstructured clinical information from electronic health records. Subsequently, the framework fine-tunes large language models using established machine learning frameworks including Hugging Face, PyTorch, and Weights & Biases, with Bayesian hyperparameter optimization to enhance model performance. Finally, the trained models generate clinical risk scores that are automatically integrated back into FHIR RiskAssessment resources, enabling seamless deployment within existing clinical workflows and electronic health record systems.

Our production environment runs an HL7 FHIR Release 4 (R4) server. All FHIR resources extracted and used in this study conform to the German Medical Informatics Initiative (MII) implement ation guides for the respective profiles, with the exception of RiskAssessment, DiagnosticReport and ServiceRequest, which were used as core R4 resources without MII-specific profiling. The referenced MII profiles are publicly documented in the MII implementation guides repository (Simplifier, MII Guides: https://simplifier.net/organization/koordinationsstellemii/∼guides).

### Tasks, model, and cohort selection

In order to demonstrate the framework’s capabilities, four classification tasks were evaluated: two single-label tasks and two multi-label tasks. The single-label tasks included predicting the risk of a patient being readmitted to the hospital within 30 days of discharge, and in-hospital mortality, defined as whether a patient dies during their hospital stay. The multi-label tasks involved predicting imaging studies performed within the first 24 hours of hospital admission and ICD-10-GM codes after the first day of admission until the end.

We utilized the GeBERTa base and large model^20^ due to their proven effectiveness in German clinical text processing, making it well-suited for understanding and extracting relevant information from the German electronic health records. However, the approach is sufficiently flexible to permit the use of other models, including English ones, by simply modifying the model name in the configuration, as the model is pulled from the public Huggingface model repository and used in the pipeline as demonstrated in Figure 1. The models were trained using seven years of continuous retrospective data, spanning inpatient settings from January 2018 to December 2024. This extensive dataset enabled robust model development and evaluation.

### Sampling and training strategy

**Figure 2.**
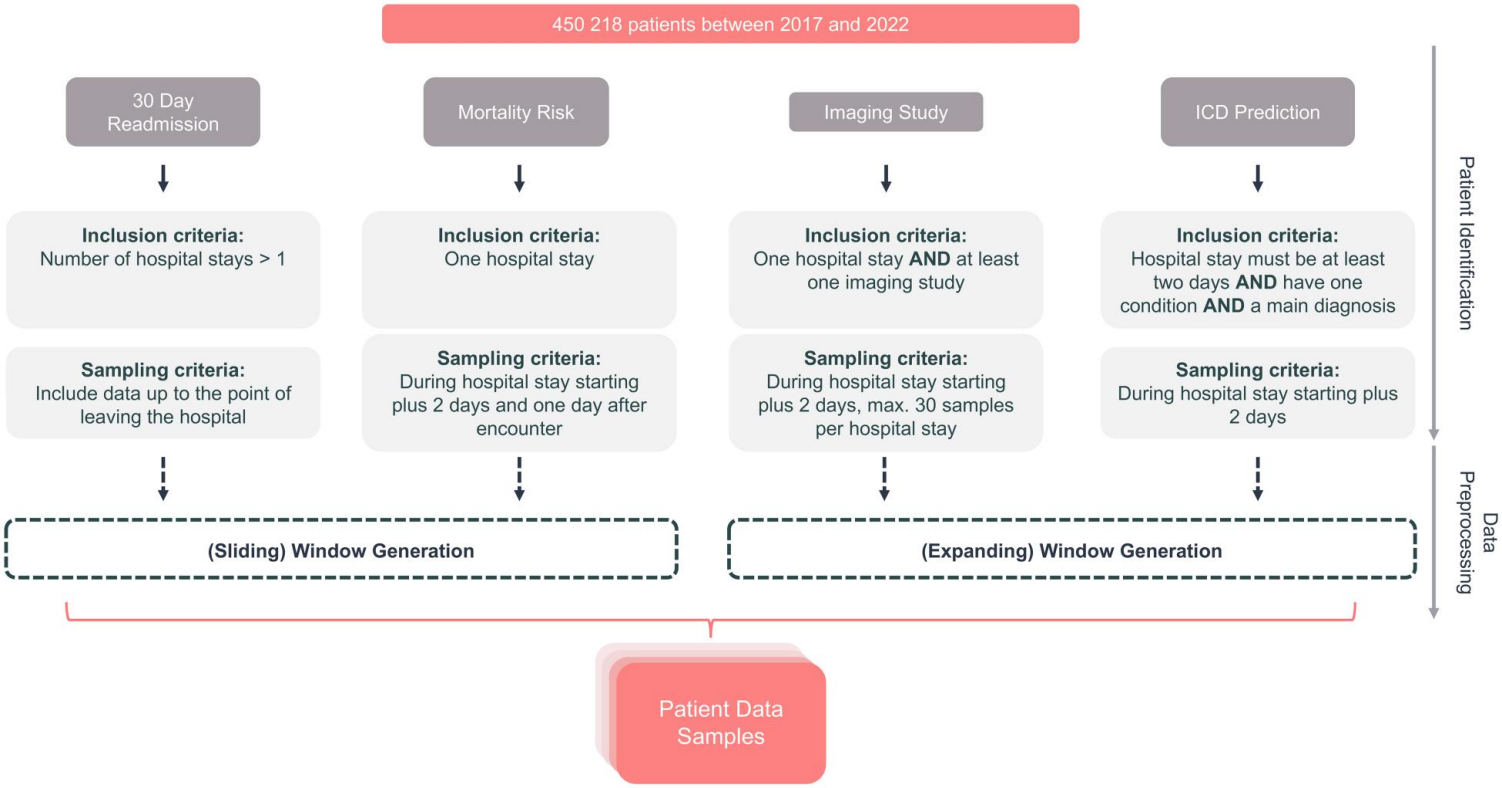
Cohort selection and data sampling workflow. Overview of inclusion criteria and sampling strategies for four prediction tasks, illustrating patient identification, cohort selection, and data preprocessing using sliding and expanding window methods.

As shown in Figure 2, the dataset comprised 450 218 patients admitted for inpatient stays between 2017 and 2022. Task-specific inclusion criteria were applied to define cohorts: Firstly, for the 30-day readmission task, only patients with multiple hospital stays. Secondly, for mortality prediction, all patients with a single hospital admission were included. Thirdly, the imaging study prediction cohort required at least one imaging study during the hospital stay. Finally, the ICD prediction cohort included patients with hospital stays of at least two days and at least one recorded condition and a main diagnosis. The application of these criteria ensured that the data were both relevant and representative for each task.

**Figure 3.**
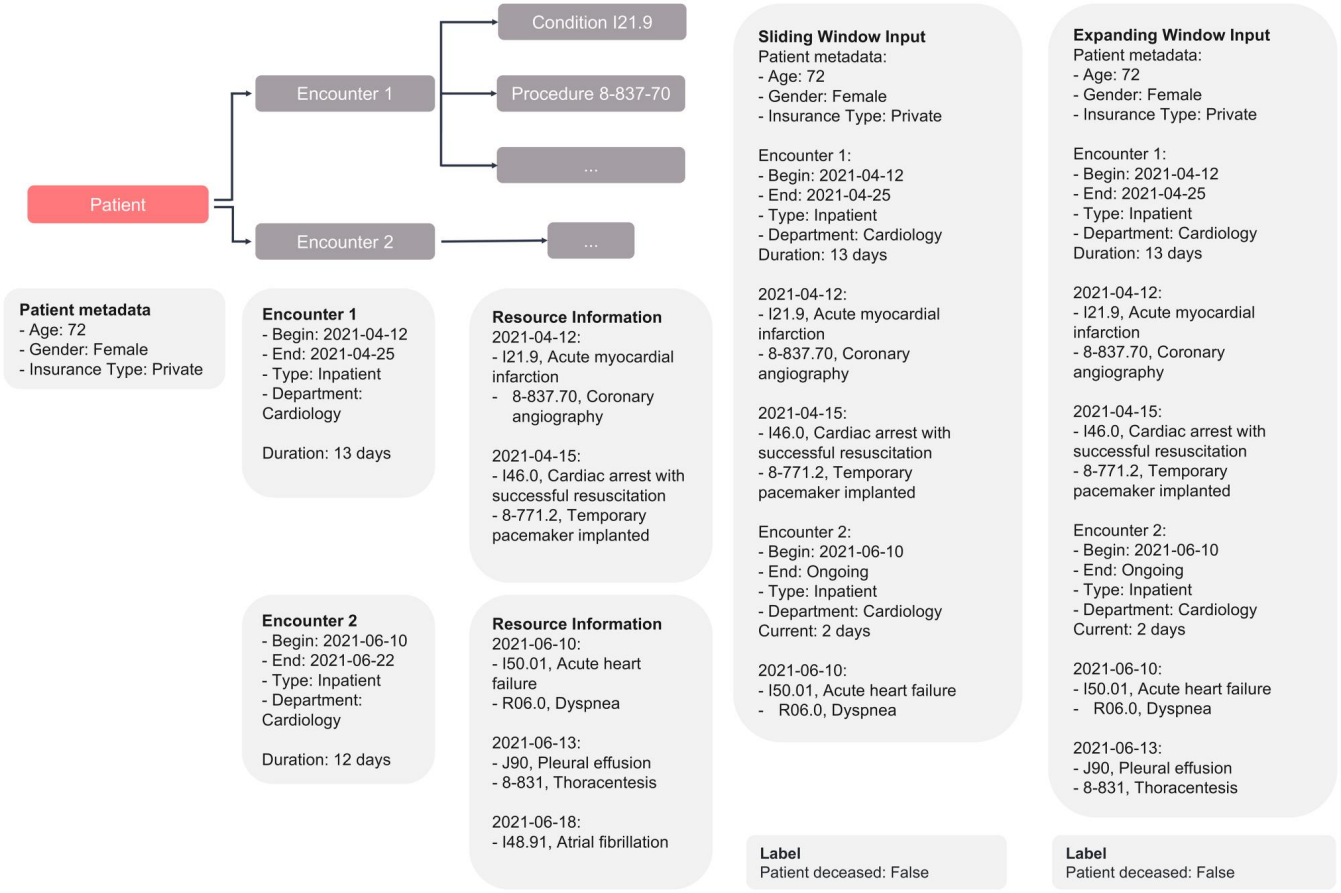
Sliding vs expanding window data generation. Illustrating two sampling methods for longitudinal patient data. The sliding window treats each hospital encounter (eg, separate 13- and 12-day admissions) as independent, capturing distinct clinical episodes with fixed metadata. The expanding window, on the other hand, continuously updates a single hospitalization (eg, over 12 days), integrating unique daily events to reflect evolving conditions. The utilization of dashed timelines and procedures (eg, angiography) illustrates the manner in which each approach structures data for mortality prediction, thereby highlighting isolated snapshots as opposed to continuous progression.

This study utilized two data sampling strategies: the sliding window and expanding window approaches, to structure longitudinal patient data across a range of downstream tasks. In the sliding window generation method, each hospital encounter (eg, inpatient stays) was treated as an independent sample. The window advanced sequentially from one encounter to the next, ensuring non-overlapping temporal segments. This approach isolated distinct clinical episodes, capturing unique characteristics and outcomes of individual hospitalizations. Conversely, the expanding window generation method generated multiple samples per encounter by iteratively incorporating data from each day of hospitalization. Starting from admission, the window expanded on a daily basis to include cumulative clinical information, enabling a temporally progressive view of the patient’s condition. This method is particularly suited for modeling evolving clinical scenarios, as it retains historical data while integrating new observations. Both strategies were applied to the downstream tasks, with Figure 3 illustrating their structural differences. The sliding window emphasized discrete encounters (eg, two separate cardiology admissions), while the expanding window simulated real-time updates within a single ongoing admission (eg, daily updates during a 12-day stay).

For the training, the Hugging Face Transformers library with PyTorch as a backend framework was utilized. The process was orchestrated through a command-line interface that handled both single-label and multi-label classification tasks. The fine-tuning hyperparameters were optimized using Weights & Biases (W&B) sweeps with Bayesian optimization. The sweep configuration, which defined the search space for various data inclusion parameters, such as the use of imaging studies, procedures, diagnostic reports, observations, medications, and other FHIR resources, was instrumental in this process. The objective of the optimization was to minimize the evaluation loss, with a cap of 50 runs per sweep on the GeBERTa-base model with a maximum of 100 000 samples for each run. The training process was integrated with W&B for the purpose of experiment tracking, with key metrics including precision, recall, and F1-scores being logged. Additionally, parameter importance and correlation metrics were logged using W&B’s parameter importance panel. For the final model, GeBERTa-large was trained on the minimal evaluation loss model of the sweep on all samples. Model checkpoints were saved during the training process, and the model that demonstrated the best performance was selected based on validation metrics for final deployment.

### Deployment pipeline

In order to facilitate the deployment of the model, a clinical decision support pipeline was implemented. This pipeline processes patient FHIR data in real-time, thereby generating risk predictions. The deployment architecture consists of three main components: model prediction, risk score calculation, and pushing the results to the local FHIR server, where it can be utilized. The prediction pipeline first extracts and preprocesses relevant FHIR resources from incoming patient data using the same preprocessing steps applied during training. The fine-tuned model then generates predictions, which are converted into risk scores ranging from 0 to 1. These scores are then automatically mapped back to the FHIR format as RiskAssessment (https://hl7.org/fhir/R4/riskassessment.html) resources, thus ensuring improvement of healthcare interoperability standards. A sample prediction can be found in the Figure S1. The stored predictions on the FHIR server act as an audit trail of predictions and allow continuous monitoring of the model’s performance. Risk assessments are delivered to FHIR, which enables seamless integration to incorporate the model’s predictions into the doctor’s decision-making process while maintaining their established workflows.

## Results

### Pipeline adaptability and expansion

The pipeline is orchestrated through a centralized command-line interface (CLI), managing data extraction, preprocessing, and model training. This interface allows researchers fine-grained control via command-line arguments and configuration files, facilitating modifications without code adjustments.

The pipeline demonstrates adaptability through four primary adaptation strategies:

1. Configuration-driven customization. The configuration file allows researchers to dynamically define:
  - Selection of attributes for contextual analysis (eg, diagnostic codes or patient demographics).
  - Temporal columns for chronological sorting (eg, patient admission dates).
  - Deduplication criteria for columns.
2. Expandable architecture for new tasks. This approach separates shared data-handling logic from task-specific requirements:
  - Single-label classification: Demonstrated in mortality prediction, where models process historical encounters to predict binary mortality risk.
  - Multi-label classification: Implemented for ICD-10 code prediction, generating simultaneous predictions across multiple diagnostic codes.
  Both tasks reuse core preprocessing workflows while customizing label generation and temporal constraints, ensuring consistency without sacrificing task-specific needs.
3. Systematic hyperparameter optimization. Integration with Weights & Biases (W&B) enables structured exploration of model configurations through sweep functionalities:
  - Discrete parameters: Selection of FHIR resources (eg, excluding imaging reports to assess performance impact).
  - Continuous parameters: Learning rate ranges or dropout probabilities.
4. Clinical workflow integration. The outputs of the model are converted to FHIR RiskAssessment resources, thus ensuring interoperability with EHR systems. This standardization allows clinicians to view predictions directly within patient records, thereby bridging the gap between analytics and care delivery.

The pipeline is accessible on GitHub at: https://github.com/UMEssen/fhir-former.

### Patient characteristics

After sampling, the study cohort included a total of 408 267 unique patients admitted between 2017 and 2022, who were selected according to task-specific inclusion criteria. Patients were evaluated across four distinct predictive tasks: 30-day hospital readmission (*n* = 295 605), in-hospital mortality (*n* = 408 267), imaging study prediction (*n* = 111 842), and ICD-10 code classification (54 151 patients).

The age distribution varied across tasks, with median ages ranging from 51.4 years (readmission) to 60.8 years (ICD code prediction). The age distribution exhibited a predominance of patients aged 65-99 years in multi-label prediction tasks (37.9% in imaging studies, 47.8% in ICD codes), while younger cohorts were more represented in binary prediction tasks. Female patients constituted approximately 50% of each task cohort (51.3% mortality, 50.0% readmission, 49.5% imaging, and 45.3% in ICD code prediction).

With regard to insurance coverage, the majority of patients had statutory health insurance, accounting for approximately three-quarters of each cohort. Mortality rates differed across tasks, with the highest observed in the imaging study prediction task (11.3%). Patient characteristics for each task are summarized in Table 1.

**Table 1.** Demographic and clinical characteristics of the patient cohort.

Characteristic	Mortality	Readmission	Image	Icd
Age group: 18-39	90 204 (22.1%)	59 345 (20.1%)	18 344 (16.4%)	6513 (12.0%)
Age group: 40-64	144 409 (35.4%)	106 212 (35.9%)	41 079 (36.7%)	20 031 (37.0%)
Age group: 65-99	110 676 (27.1%)	90 677 (30.7%)	42 396 (37.9%)	25 889 (47.8%)
Age group: <18	62 910 (15.4%)	39 314 (13.3%)	10 004 (8.9%)	1705 (3.1%)
Age group: ≥100	66 (0.0%)	55 (0.0%)	17 (0.0%)	13 (0.0%)
Age mean ± SD	47.3 ± 24.4	49.6 ± 24.2	54.6 ± 22.5	60.8 ± 19.3
Age median (IQR)	51.4 (29.0-66.4)	54.4 (31.7-68.4)	59.2 (39.6-71.4)	64.1 (51.4-75.1)
Age range	0.0-121.1	0.0-117.5	0.0-108.7	0.0-105.9
Average duration (days)	95.29	121.3	100.37	173.4
Classification type	Binary	Binary	Multi-class	Multi-class
Insurance: gesetzlich	314 365 (77.0%)	223 366 (75.6%)	81 501 (72.9%)	38 645 (71.4%)
Insurance: privat	17586 (4.3%)	17 048 (5.8%)	8142 (7.3%)	4472 (8.3%)
Insurance: unbekannt	76316 (18.7%)	55 191 (18.7%)	22 199 (19.8%)	11 034 (20.4%)
Mortality	15974 (3.9%)	15 679 (5.3%)	126 44 (11.3%)	5533 (10.2%)
Number of patients	40 8267	295 605	111842	54 151
Number of samples	1 571 737	1 318 216	600781	146 590
Sex: Female	209 268 (51.3%)	147 850 (50.0%)	55 332 (49.5%)	24 542 (45.3%)
Sex: Male	195 804 (48.0%)	145 194 (49.1%)	55 096 (49.3%)	29 157 (53.8%)
Sex: Unknown	3195 (0.8%)	2561 (0.9%)	1414 (1.3%)	452 (0.8%)

### Intermediate modeling results and feature contribution analysis

For each task, we executed Bayesian optimization sweeps to identify optimal combinations of FHIR resources and training parameters. Tables S1-S4 summarize the top-performing configurations for imaging study, ICD code, readmission, and mortality prediction tasks, respectively. For example, in imaging study prediction (Table S1), the best-performing model incorporated Episode of Care achieving a macro validation F1-score of 0.57 Similarly, for the readmission task (Table S3), the inclusion of Biologically Derived Product (bdp), Episode of Care (eoc), Medication (med), Observation (obs), and Procedure (prc), yielding an validation F1-score of 0.68. The results of this study demonstrate the task-specific relevance of specific FHIR resources. For example, the results indicate that the resource med is relevant for mortality prediction, and bdp is relevant for the icd prediction task.

To quantify the contribution of individual FHIR resources, we analyzed feature importance and correlation coefficients across tasks (Table S5). For imaging prediction, Service Request showed the highest importance (0.265) but strongest inverse correlation (−0.511), reflecting its dual role in workflow prioritization. Medication had the strongest positive correlation (0.432), linking prescriptions to imaging needs. In ICD coding, Procedure importance (0.224) dominated, while Imaging Study correlation (−0.137) indicated limited standalone utility. For readmission, Condition exhibited maximal importance (0.29) and correlation (0.534), emphasizing comorbidities, whereas Episode of Care had minimal impact (0.017). For mortality, Procedure showed the strongest positive correlation (0.252), suggesting links to critical interventions, while Service Request had the most negative correlation (−0.268), aligning with end-of-life care trends. These extremes show that FHIR-Former can uncover important patterns specific to each task, balancing strong performance with clear explanations of which clinical features matter most.

### Final model performance

As illustrated in Table 2, the key performance metrics of FHIR-Former demonstrate its efficacy in two clinical prediction tasks. In the context of the 30-day readmission task, the system attained an accuracy of 73.3% and an F1-score of 70.9%, with comparable performance metrics observed on the test set (72.9%, 70.7%). In the in-hospital mortality task, the best evaluation run reached an accuracy of 93.6% with an F1-score of 55.2%, while the test performance showed an accuracy of 88.1% and an F1-score of 51.8%.

**Table 2.** Downstream task readmission and mortality.

Downstream task	Split	Accuracy	Precision	Recall	F1	ROC AUC
Readmission	Validation	0.7333	0.7049	0.715	0.7087	0.715
Readmission	Test	0.729	0.7024	0.7158	0.7067	0.7158
Mortality	Validation	0.9358	0.5369	0.7537	0.5519	0.7537
Mortality	Test	0.8806	0.5251	0.7841	0.5178	0.7841

As demonstrated in Table 3, for the multi-label imaging study task, the most successful evaluation run attained an accuracy of 85% with a macro F1 score of 60%, whereas the test run yielded an accuracy of 86% and a macro F1 score of 61%. In the ICD prediction task, the final model recorded an accuracy of 94%, a top 10 accuracy of 90%, yet a macro F1 score of 50%. However, the test performance was consistent, showing an accuracy of 94%, a top 10 accuracy of 89% and a macro F1 score of 48%. The final resource configurations used for each task correspond to the best-performing models shown in Tables S1-S4.

**Table 3.** Downstream task image and ICD.

Downstream task	Split	Accuracy	Macro precision	Macro recall	Macro F1	Top10 accuracy	Top10 Macro precision	Top10 Macro recall	Top10 Macro F1
Image	Validation	0.85	0.71	0.55	0.6	0.85	0.71	0.55	0.6
Image	Test	0.86	0.72	0.55	0.61	0.86	0.72	0.55	0.61
ICD	Validation	0.94	0.71	0.43	0.5	0.9	0.73	0.6	0.63
ICD	Test	0.94	0.71	0.42	0.48	0.89	0.71	0.59	0.62

## Discussion

The FHIR-Former package addresses critical limitations in healthcare predictive modeling by offering configurability and usability. Its dynamic adaptation to diverse FHIR resources (eg, medications, lab values, clinical notes) eliminates the need for manual feature engineering, thereby enabling seamless integration of both structured and unstructured data. The software’s modular architecture, supported by a command-line interface, simplifies workflows into discrete phases (extract, sampling, training), thus lowering barriers for non-technical users. Researchers are able to rapidly prototype new single- or multi-label tasks without modifying the core infrastructure. Furthermore, compatibility with existing FHIR servers ensures interoperability, while the checkpoint-based training design demonstrates the potential for federated learning across institutions.

Conventional predictive models depend on labour-intensive extract, transform, load pipelines and static input configurations, which hinder scalability and generalizability.^10^ For instance, Rajkomar et al^10^ achieved strong performance but required institution-specific FHIR customization, while ClinicalBERT^17^ focused narrowly on unstructured text. In contrast, FHIR-Former automates data harmonization and dynamically adapts to institutional FHIR variations, reducing preprocessing tasks. In contrast to frameworks that are limited to predefined tasks (eg, Cardea^18^), FHIR-Former’s configurable architecture supports rapid task expansion, as shown by its successful application to four distinct clinical predictions.

FHIR-Former demonstrated promising predictive performance for 30-day readmissions (F1-score: 70.7%, accuracy: 72.9%) compared to existing models. A systematic review of 73 risk prediction models for 28-day or 30-day unplanned hospital readmissions reported c-statistics ranging from 0.21 to 0.88, with only two models exceeding 0.8, and many exhibiting moderate performance (0.55-0.70).^21^ Thus, FHIR-Former’s performance indicates improved precision and recall balance relative to these benchmarks. In contrast, FHIR-Former’s predictive performance for in-hospital mortality (F1-score: 51.8%, accuracy: 88.1%) was lower compared to results reported by Choi et al. (2022), where models using electronic health records achieved higher F1-scores (84%). However, FHIR-Former was evaluated on a broader patient population, beyond intensive care units.^22^

A direct comparison with existing models on shared datasets would strengthen the evaluation of FHIR-Former’s performance. However, such comparisons present methodological challenges in the current study. The benchmark datasets from the stated studies are generally not publicly available, and when they are, they are not provided as FHIR bundles, which limits reproducibility and direct comparison. Additionally, existing state-of-the-art models for readmission and mortality prediction (eg, logistic regression, XGBoost, SVM) are designed exclusively for structured data inputs. Comparing models with fundamentally different input modalities would require discarding the multimodal capabilities that constitute a core contribution of our framework. Future studies should establish standardized benchmarks that accommodate multimodal clinical prediction frameworks to enable more comprehensive performance evaluations. By automating risk assessment, the framework has the capacity to streamline resource allocation, for example, by prioritizing high-risk patients for post-discharge monitoring or by tailoring imaging study workflows. The incorporation of risk scores into FHIR servers in real-time ensures the relevance of the system to clinical practice, enabling healthcare providers to act on predictions without disrupting existing workflows. The translation of these capabilities to clinical practice may result in a reduction in readmission rates, earlier interventions, and more personalized care pathways.

Although FHIR-Former’s automated approach using BERT-based language models eliminates the need for manual feature engineering and achieves strong predictive performance, it reduces model interpretability compared to traditional feature-based approaches. Although Table S5 provides resource importance analyses that demonstrate the varying contributions of FHIR resources across tasks, the transformer architecture inherently functions as a “black box,” which limits clinicians ability to understand the reasoning behind individual predictions. The framework currently supports ten core FHIR resources (Patient, Encounter, Condition, Procedure, Observation, ImagingStudy, DiagnosticReport, EpisodeOfCare, ServiceRequest, and Medication), with each resource implemented as a dedicated build method. Expanding to additional resource types requires implementing corresponding extraction methods following the established pattern. The pipeline’s flexibility extends to accommodating institution-specific FHIR implementations through configurable parameters and custom field mappings. However, variability in local coding practices and resource completeness may necessitate institution-specific tuning of extraction logic, particularly for resources with complex nested structures or non-standard extensions. FHIR-Former’s architecture demonstrates model agnosticism through its use of Hugging Face’s transformer library, this enables integration of alternative encoder and decoder-based language models by updating the model checkpoint configuration. The framework’s task expansion capabilities are exemplified by the modular dataset builders. New prediction tasks can be implemented by creating task-specific builders that define custom sampling strategies, label extraction logic, and feature generation methods. The sliding and expanding window approaches for longitudinal data sampling provide templates for temporal modeling tasks, while the configurable preprocessing pipeline accommodates different label formats and class distributions. Task-specific considerations, such as class imbalance handling in multi-label scenarios and stratified sampling strategies, are encapsulated within individual task implementations.

Several practical challenges may arise when deploying FHIR-Former across diverse clinical settings. Local FHIR implementations exhibit significant variations in resource completeness, coding systems, and data quality, necessitating institution-specific validation rules and quality assurance protocols. For new clinical sites, task-specific models typically require retraining due to institutional differences in available FHIR resources, data content variations, and local coding practices that affect model performance and generalizability. The framework’s computational requirements for model training and inference demand substantial GPU resources, where resource-constrained environments may require model distillation, quantization, or cloud deployment strategies, leveraging the framework’s configurable batch processing capabilities. Continuous performance monitoring is essential to detect data drift, and establish retraining protocols. Although the framework generates standardized RiskAssessment resources, successful clinical deployment requires careful consideration of alert fatigue, workflow timing, and integration overhead within existing clinical decision support infrastructures. This pipeline is intended for research purposes only and requires further validation and compliance review before deployment in clinical settings to support medical decision-making.

The study currently focuses on inpatient settings, which limits the generalizability of the results to outpatient or ambulatory settings. Also the models that were trained are dependent on the data structure and resources of our infrastructure, therefore new models should be trained on data that represents the institution’s resources and data structures. Additionally, the framework’s performance is dependent on the quality and completeness of FHIR resources within a single institution, which may introduce biases from data entry errors or missing longitudinal records (eg, external doctor visits). While the pipeline accommodates diverse FHIR implementations, variability in local coding practices could still necessitate task-specific tuning for the data extraction step.

The inclusion of data from outpatient settings, coupled with the integration of multimodal data (eg, wearables, genomics), could enhance the predictive capabilities of the downstream tasks. For instance, models like Llava^23^ combine transformer-based image embeddings with language model embeddings, demonstrating the potential of multimodal approaches in improving predictive performance. Following the operationalization of the prediction pipeline, prospective studies should evaluate its real-world impact by validating risk scores in live clinical workflows and measuring improvements in decision-making efficiency and patient outcomes. Finally, the utilization of transformer decoder models has the potential to facilitate generative tasks, such as synthesizing clinical notes or recommending treatment plans.

The FHIR-Former package represents a substantial advancement in the realm of scalable, interoperable healthcare analytics. By integrating the standardization offered by FHIR with the flexibility of LLMs, it serves to mitigate the persistent challenges posed by data heterogeneity and the necessity of manual preprocessing. Despite the presence of ongoing limitations, the open-source availability and modular design of FHIR-Former position it as a foundational tool for the advancement of predictive medicine, thereby serving to bridge the gap between AI and real-world clinical workflows.

## Supplementary Material

ocaf165_Supplementary_Data

## Data Availability

The data underlying this article will be shared on reasonable request to the corresponding author.

## Code availability

The underlying code for this study is available in FHIR-Former and can be accessed via this link https://github.com/UMEssen/fhir-former.
