# Supplementary material for: FHIR-Former: enhancing clinical predictions through Fast Healthcare Interoperability Resources and large language models
Source: J Am Med Inform Assoc. 2025 Oct 13;32(12):1793–801. doi: 10.1093/jamia/ocaf165 (PMC12646377; doi:10.1093/jamia/ocaf165)
Supplement: ocaf165_Supplementary_Data [file ocaf165_supplementary_data.docx]

# SUPPLEMENT TO

Improving Clinical Decision Making with FHIR and Large Language Models

Merlin Engelke^1,2^, Giulia Baldini^1,2^, Jens Kleesiek^1,3,4^, Felix Nensa^1,2^, Amin Dada^1^

^1^ Institute for Artificial Intelligence in Medicine, University Medicine Essen, Essen, Germany

^2^ Institute of Diagnostic and Interventional Radiology and Neuroradiology, University Medicine Essen, Essen, Germany

^3^ Faculty of Computer Science and Medical Faculty, University of Duisburg-Essen, Essen, Germany

^4^ TU Dortmund University, Department of Physics, Dortmund, Germany

## Supplementary Figures

| {  "resourceType": "Bundle",  "meta": {  "extension": [  {  "url": "https://uk-essen.de/ship/pagination-notice",  "valueString": "Pagination may return incorrect results. Specify the '_sort' parameter for correct results."  }  ]  },  "type": "searchset",  "total": 1,  "link": [  {  "relation": "self",  "url": "/app/FHIR/r4/RiskAssessment?_id=deidentified"  },  {  "relation": "first",  "url": "/app/FHIR/r4/RiskAssessment?_id=deidentified&_shipPagination=eyJvZmZzZXQiOjB9"  }  ],  "entry": [  {  "fullUrl": "https://ship.ume.de/app/FHIR/r4/RiskAssessment/deidentified",  "resource": {  "resourceType": "RiskAssessment",  "id": "deidentified-riskassessment-1",  "meta": {  "extension": [  {  "url": "http://fhirformer.org/extension-source-version",  "valueString": "0.0.1"  }  ],  "versionId": "version-1",  "lastUpdated": "2025-03-29T11:50:20.425+00:00",  "source": "urn:fhirformer:ml",  "profile": [  "https://fhirformer.org/StructureDefinition/ml-prediction"  ]  },  "language": "de",  "status": "final",  "method": {  "coding": [  {  "system": "https://ship.ume.de/fhir/ml/models",  "version": "1.0",  "code": "**ikim-uk-essen_geberta-large-image**",  "display": "FHIRFormer ML Model for imaging prediction"  }  ],  "text": "ML Model: example-model"  },  "subject": {  "reference": "Patient/deidentified",  "type": "Patient",  "display": "Deidentified Patient"  },  "occurrenceDateTime": "2025-03-29T11:50:20.401+00:00",  "basis": [  {  "reference": "Encounter/deidentified",  "type": "Encounter",  "display": "Encounter for deidentified patient"  },  {  "reference": "Condition/example-condition",  "type": "Condition",  "display": "Example Condition"  },  {  "reference": "Observation/example-observation",  "type": "Observation",  "display": "Example Observation"  }  ],  "prediction": [  {  "probabilityDecimal": 0.8515367674827576,  "whenPeriod": {  "start": "2025-03-29T11:10:27.665+00:00",  "end": "2025-03-30T11:10:27.665+00:00"  },  "qualitativeRisk": {  "coding": [  {  "system": "https://ship.ume.de/fhir/ml/imaging-modality",  "code": "CT",  "display": "Predicted Imaging Modality: CT",  "version": "1.0"  }  ],  "text": "Predicted modality: CT"  }  }  ],  "note": [  {  "text": "Deidentified input string containing all resoruces."  }  ]  },  "search": {  "mode": "match"  }  }  ] } |
| --- |

**Supplemental Figure 1:** Example Risk Assessment Profile for task readmission.

This supplementary figure presents a representative example of a FHIR RiskAssessment resource generated by the FHIR-Former pipeline following the prediction of a clinical outcome. The resource illustrates how model-generated risk scores are integrated into the standardized FHIR format, ensuring compatibility with clinical decision support systems and EHR environments.In this particular instance, the fine tuned machine learning model ikim-uk-essen_geberta-base-image assessed risk for a patient using data from an Encounter, a Condition, and an Observation. The model produced a probability of approximately 0.85 for a clinical event and indicated CT as the predicted imaging modality.

The generated FHIR resource contains all required contextual elements without additional post-processing. Key attributes include: patient identifiers (subject.reference to "Patient/deidentified"), method codes (system, version, code, and display for the ML model), prediction details (prediction score, validity), model input data from raw resources (Encounter,Condition, and Observation) and input string (note[0].text to "Deidentified input string containing all resoruces."). This resource structure demonstrates the integration of AI-generated predictions into clinical workflows using standard-compliant FHIR formatting.

## Supplementary Tables

Abbreviations:

bdp – Biologically Derived Product

con – Condition

drp – Diagnostic Report

eoc – Episode of Care

img – Imaging Study

med – Medication

obs – Observation

prc – Procedure

srv – Service Request

mprc – Macro Precision

mrec – Macro Recall

mf1 / f1 – Macro F1-score

acc – Accuracy

auc – Area Under the ROC Curve (AUC-ROC)

**Note**: Metric names like f1, mprc, and mrec may appear with or without the m prefix. In this context, f1 = mf1, prc = mprc, and rec = mrec, all referring to macro-averaged values. These should be treated as equivalent unless otherwise specified.

**Supplement Table 1**: Top 20 image classification runs ranked by evaluation loss.

| bdp | con | drp | eoc | med | obs | prc | srv | loss | acc | mprc | mrec | mf1 |
| --- | --- | --- | --- | --- | --- | --- | --- | --- | --- | --- | --- | --- |
|  |  |  | ✓ |  |  |  |  | 0.343 | 0.84 | 0.68 | 0.52 | 0.57 |
|  | ✓ |  | ✓ |  |  | ✓ | ✓ | 0.350 | 0.84 | 0.73 | 0.51 | 0.58 |
| ✓ | ✓ | ✓ | ✓ |  |  | ✓ | ✓ | 0.353 | 0.84 | 0.71 | 0.52 | 0.59 |
| ✓ | ✓ | ✓ |  |  |  |  | ✓ | 0.353 | 0.84 | 0.68 | 0.53 | 0.58 |
|  |  | ✓ |  | ✓ |  | ✓ | ✓ | 0.355 | 0.84 | 0.68 | 0.53 | 0.57 |
|  | ✓ | ✓ |  | ✓ |  |  | ✓ | 0.358 | 0.83 | 0.68 | 0.52 | 0.56 |
|  |  |  | ✓ |  |  |  | ✓ | 0.359 | 0.84 | 0.71 | 0.51 | 0.57 |
| ✓ | ✓ | ✓ | ✓ | ✓ |  | ✓ | ✓ | 0.360 | 0.84 | 0.68 | 0.53 | 0.59 |
| ✓ | ✓ |  | ✓ |  |  |  |  | 0.362 | 0.84 | 0.66 | 0.5 | 0.55 |
| ✓ |  |  |  |  |  | ✓ |  | 0.365 | 0.84 | 0.67 | 0.47 | 0.53 |
| ✓ | ✓ |  |  | ✓ |  |  | ✓ | 0.365 | 0.84 | 0.68 | 0.5 | 0.56 |
|  |  |  |  | ✓ | ✓ | ✓ | ✓ | 0.369 | 0.82 | 0.65 | 0.48 | 0.53 |
|  | ✓ |  | ✓ |  | ✓ |  | ✓ | 0.369 | 0.83 | 0.65 | 0.47 | 0.52 |
|  | ✓ |  |  | ✓ | ✓ |  | ✓ | 0.371 | 0.83 | 0.69 | 0.47 | 0.53 |
|  | ✓ |  |  |  |  |  |  | 0.372 | 0.83 | 0.67 | 0.48 | 0.53 |
| ✓ |  | ✓ | ✓ |  |  |  |  | 0.372 | 0.83 | 0.65 | 0.48 | 0.53 |
| ✓ |  | ✓ |  |  |  | ✓ |  | 0.373 | 0.83 | 0.65 | 0.48 | 0.54 |
| ✓ |  | ✓ | ✓ | ✓ | ✓ | ✓ | ✓ | 0.375 | 0.83 | 0.69 | 0.45 | 0.52 |
| ✓ |  |  | ✓ | ✓ |  | ✓ |  | 0.376 | 0.83 | 0.61 | 0.47 | 0.51 |
| ✓ | ✓ |  | ✓ | ✓ |  |  | ✓ | 0.377 | 0.83 | 0.69 | 0.48 | 0.55 |

**Note**: All scores are evaluation metrics. Imaging study was always used as it is part of the label.

**Supplement Table 2**: Top 20 ICD classification runs ranked by evaluation loss.

| bdp | drp | eoc | img | med | obs | prc | srv | loss | acc | mprc | mrec | mf1 |
| --- | --- | --- | --- | --- | --- | --- | --- | --- | --- | --- | --- | --- |
| ✓ |  |  |  |  |  |  |  | 0.169 | 0.94 | 0.7 | 0.5 | 0.57 |
| ✓ | ✓ | ✓ | ✓ |  |  |  |  | 0.173 | 0.94 | 0.61 | 0.52 | 0.55 |
|  |  |  |  |  |  |  |  | 0.175 | 0.94 | 0.69 | 0.52 | 0.58 |
|  |  | ✓ | ✓ |  |  | ✓ |  | 0.186 | 0.93 | 0.64 | 0.46 | 0.51 |
| ✓ |  |  |  |  |  | ✓ |  | 0.186 | 0.93 | 0.7 | 0.47 | 0.54 |
|  |  |  | ✓ |  |  |  | ✓ | 0.187 | 0.93 | 0.62 | 0.48 | 0.53 |
| ✓ |  | ✓ |  | ✓ |  | ✓ | ✓ | 0.194 | 0.93 | 0.59 | 0.47 | 0.51 |
| ✓ | ✓ |  |  | ✓ |  | ✓ | ✓ | 0.195 | 0.94 | 0.58 | 0.48 | 0.51 |
|  | ✓ | ✓ | ✓ |  | ✓ | ✓ | ✓ | 0.196 | 0.93 | 0.6 | 0.41 | 0.45 |
| ✓ | ✓ | ✓ | ✓ |  | ✓ | ✓ |  | 0.196 | 0.93 | 0.56 | 0.39 | 0.44 |
| ✓ |  |  |  | ✓ |  |  | ✓ | 0.197 | 0.93 | 0.61 | 0.45 | 0.5 |
|  | ✓ | ✓ | ✓ | ✓ |  |  | ✓ | 0.199 | 0.93 | 0.57 | 0.45 | 0.48 |
|  | ✓ |  | ✓ |  | ✓ | ✓ | ✓ | 0.199 | 0.93 | 0.55 | 0.37 | 0.43 |
|  |  | ✓ |  |  | ✓ | ✓ | ✓ | 0.199 | 0.93 | 0.54 | 0.39 | 0.43 |
| ✓ | ✓ |  |  |  | ✓ | ✓ |  | 0.201 | 0.93 | 0.51 | 0.39 | 0.43 |
| ✓ | ✓ | ✓ |  |  |  |  | ✓ | 0.203 | 0.93 | 0.61 | 0.46 | 0.51 |
|  |  |  | ✓ |  | ✓ | ✓ |  | 0.203 | 0.93 | 0.54 | 0.35 | 0.41 |
|  |  |  |  |  | ✓ | ✓ | ✓ | 0.206 | 0.93 | 0.56 | 0.39 | 0.43 |
| ✓ | ✓ |  |  |  |  | ✓ | ✓ | 0.206 | 0.93 | 0.59 | 0.45 | 0.51 |
| ✓ | ✓ |  | ✓ | ✓ | ✓ |  |  | 0.206 | 0.93 | 0.56 | 0.4 | 0.45 |

**Note**: All scores are evaluation metrics. Condition was always used as it is part of the label.

**Supplement Table 3**: Top 20 readmission classification runs ranked by evaluation loss.

| bdp | con | drp | eoc | img | med | obs | prc | srv | loss | acc | prc | rec | f1 | auc |
| --- | --- | --- | --- | --- | --- | --- | --- | --- | --- | --- | --- | --- | --- | --- |
| ✓ |  |  | ✓ |  | ✓ | ✓ | ✓ |  | 0.615 | 0.71 | 0.68 | 0.68 | 0.68 | 0.68 |
|  |  |  |  |  |  |  |  | ✓ | 0.637 | 0.72 | 0.69 | 0.69 | 0.69 | 0.69 |
|  | ✓ | ✓ |  |  | ✓ | ✓ | ✓ |  | 0.640 | 0.67 | 0.65 | 0.68 | 0.65 | 0.68 |
|  |  |  |  |  |  |  |  |  | 0.642 | 0.68 | 0.67 | 0.68 | 0.67 | 0.68 |
|  |  |  |  |  |  |  |  |  | 0.651 | 0.7 | 0.35 | 0.5 | 0.41 | 0.5 |
| ✓ |  |  | ✓ |  |  | ✓ |  | ✓ | 0.666 | 0.7 | 0.66 | 0.67 | 0.66 | 0.67 |
|  | ✓ |  |  |  | ✓ | ✓ | ✓ |  | 0.686 | 0.71 | 0.66 | 0.68 | 0.67 | 0.68 |
|  |  |  |  |  | ✓ |  | ✓ |  | 0.692 | 0.69 | 0.66 | 0.66 | 0.66 | 0.66 |
|  |  | ✓ |  | ✓ | ✓ |  |  | ✓ | 0.721 | 0.72 | 0.68 | 0.7 | 0.69 | 0.7 |
|  |  |  |  |  |  |  |  |  | 0.734 | 0.3 | 0.15 | 0.5 | 0.23 | 0.5 |
| ✓ |  |  | ✓ | ✓ | ✓ |  | ✓ |  | 0.738 | 0.68 | 0.66 | 0.68 | 0.66 | 0.68 |
|  |  |  |  |  |  |  |  |  | 0.742 | 0.3 | 0.15 | 0.5 | 0.23 | 0.5 |
|  |  |  |  |  |  |  |  |  | 0.745 | 0.3 | 0.15 | 0.5 | 0.23 | 0.5 |
|  | ✓ |  | ✓ | ✓ |  |  |  | ✓ | 0.764 | 0.71 | 0.68 | 0.7 | 0.68 | 0.7 |
| ✓ | ✓ | ✓ | ✓ |  | ✓ | ✓ |  | ✓ | 0.766 | 0.7 | 0.67 | 0.7 | 0.67 | 0.7 |
| ✓ | ✓ | ✓ |  |  | ✓ | ✓ |  |  | 0.767 | 0.7 | 0.66 | 0.69 | 0.67 | 0.69 |
|  |  | ✓ |  |  | ✓ |  | ✓ |  | 0.773 | 0.7 | 0.66 | 0.69 | 0.67 | 0.69 |
| ✓ |  | ✓ |  | ✓ | ✓ |  | ✓ | ✓ | 0.782 | 0.7 | 0.67 | 0.69 | 0.67 | 0.69 |
| ✓ |  |  |  |  | ✓ |  | ✓ | ✓ | 0.791 | 0.69 | 0.66 | 0.68 | 0.67 | 0.68 |
|  |  | ✓ | ✓ |  |  |  | ✓ |  | 0.798 | 0.71 | 0.68 | 0.7 | 0.68 | 0.7 |

**Note**: All scores are evaluation metrics.

**Supplement Table 4**: Top 20 mortality classification runs ranked by evaluation loss.

| bdp | con | drp | eoc | img | med | obs | prc | srv | loss | acc | prc | rec | f1 | roc |
| --- | --- | --- | --- | --- | --- | --- | --- | --- | --- | --- | --- | --- | --- | --- |
| ✓ |  | ✓ | ✓ | ✓ | ✓ |  | ✓ | ✓ | 0.251 | 0.9 | 0.53 | 0.81 | 0.53 | 0.81 |
| ✓ |  |  |  |  |  |  |  | ✓ | 0.287 | 0.88 | 0.53 | 0.8 | 0.52 | 0.8 |
| ✓ |  |  |  | ✓ |  |  | ✓ | ✓ | 0.301 | 0.88 | 0.53 | 0.84 | 0.53 | 0.84 |
|  |  | ✓ | ✓ | ✓ |  | ✓ | ✓ | ✓ | 0.328 | 0.86 | 0.53 | 0.84 | 0.52 | 0.84 |
| ✓ | ✓ |  | ✓ |  |  | ✓ |  | ✓ | 0.330 | 0.88 | 0.53 | 0.82 | 0.52 | 0.82 |
|  | ✓ |  |  |  | ✓ | ✓ |  |  | 0.339 | 0.87 | 0.53 | 0.83 | 0.52 | 0.83 |
| ✓ |  |  | ✓ |  |  | ✓ |  |  | 0.341 | 0.82 | 0.52 | 0.8 | 0.5 | 0.8 |
| ✓ | ✓ | ✓ |  | ✓ | ✓ | ✓ |  | ✓ | 0.344 | 0.85 | 0.52 | 0.82 | 0.51 | 0.82 |
|  |  |  |  | ✓ | ✓ |  | ✓ |  | 0.345 | 0.85 | 0.53 | 0.79 | 0.51 | 0.79 |
|  |  |  |  |  |  | ✓ |  | ✓ | 0.346 | 0.82 | 0.52 | 0.79 | 0.49 | 0.79 |
|  |  |  | ✓ | ✓ | ✓ |  | ✓ | ✓ | 0.363 | 0.86 | 0.53 | 0.83 | 0.51 | 0.83 |
| ✓ | ✓ | ✓ |  | ✓ | ✓ |  |  |  | 0.367 | 0.84 | 0.52 | 0.81 | 0.5 | 0.81 |
| ✓ |  | ✓ |  | ✓ |  | ✓ |  | ✓ | 0.377 | 0.85 | 0.52 | 0.82 | 0.5 | 0.82 |
|  |  |  | ✓ | ✓ | ✓ | ✓ |  |  | 0.383 | 0.81 | 0.52 | 0.78 | 0.48 | 0.78 |
| ✓ | ✓ | ✓ | ✓ |  |  | ✓ |  |  | 0.384 | 0.83 | 0.52 | 0.83 | 0.5 | 0.83 |
|  | ✓ |  | ✓ | ✓ |  | ✓ |  | ✓ | 0.393 | 0.83 | 0.52 | 0.81 | 0.49 | 0.81 |
|  |  | ✓ | ✓ | ✓ |  |  | ✓ | ✓ | 0.398 | 0.84 | 0.52 | 0.84 | 0.5 | 0.84 |
|  | ✓ |  | ✓ | ✓ | ✓ | ✓ | ✓ | ✓ | 0.402 | 0.84 | 0.52 | 0.83 | 0.5 | 0.83 |
|  |  | ✓ |  |  | ✓ | ✓ | ✓ | ✓ | 0.405 | 0.85 | 0.52 | 0.82 | 0.5 | 0.82 |
|  | ✓ |  | ✓ |  | ✓ |  |  |  | 0.405 | 0.82 | 0.52 | 0.84 | 0.49 | 0.84 |

**Note**: All scores are evaluation metrics.

**Supplement Table 5**: Feature importance and correlation based on the downstream task

|  | Image | | ICD | | Readmission | | Mortality | |
| --- | --- | --- | --- | --- | --- | --- | --- | --- |
| Resource | Importance | Correlation | Importance | Correlation | Importance | Correlation | Importance | Correlation |
| Biologically Derived Product | 0.021 | 0.025 | 0.136 | -0.021 | 0.152 | 0.267 | 0.08 | 0.01 |
| Condition | 0.045 | -0.284 | * | * | 0.29 | 0.534 | 0.179 | 0.054 |
| Diagnostic Report | 0.084 | -0.132 | 0.171 | 0.127 | 0.031 | 0.283 | 0.177 | -0.132 |
| Episode of Care | 0.066 | 0.011 | 0.068 | 0.155 | 0.017 | 0.005 | 0.059 | 0.01 |
| Imaging Study | * | * | 0.05 | -0.137 | 0.019 | 0.202 | 0.108 | 0.148 |
| Medication | 0.215 | 0.432 | 0.107 | 0.274 | 0.075 | -0.22 | 0.12 | 0.117 |
| Observation | 0.231 | 0.306 | 0.132 | 0.313 | 0.144 | -0.169 | 0.109 | -0.009 |
| Procedure | 0.074 | -0.093 | 0.224 | 0.199 | 0.097 | 0.157 | 0.156 | 0.252 |
| Service Request | 0.265 | -0.511 | 0.113 | 0.087 | 0.174 | 0.057 | 0.072 | -0.268 |

* These FHIR-Resources were always activated as they were part of the label
